# Preventative tele-health supported services for early stage chronic obstructive pulmonary disease: a protocol for a pragmatic randomized controlled trial pilot

**DOI:** 10.1186/1745-6215-12-6

**Published:** 2011-01-07

**Authors:** Deborah A Fitzsimmons, Jill Thompson, Mark Hawley, Gail A Mountain

**Affiliations:** 1 Rehabilitation and Assistive Technology Group, School of Health and Related Research, University of Sheffield, Regent Court, 30 Regent Street, Sheffield, South Yorkshire, S1 4DA, UK

## Abstract

**Background:**

Chronic Obstructive Pulmonary Disease (COPD) is a prevalent debilitating long term condition. It is the second most common cause of emergency admission to hospital in the UK and remains one of the most costly conditions to treat through acute care.

Tele-health monitoring offers potential to reduce the rates of re-hospitalisation and emergency department visits and improve quality of life for people with COPD. However, the current evidence base to support technology adoption and implementation is limited and the resource implications for implementing tele-health in practice can be very high. This trial will employ tele-health monitoring in a preventative capacity for patients diagnosed with early stage COPD following discharge from hospital to determine whether it reduces their need for additional health service support or hospital admission and improves their quality of life.

**Methods/Design:**

We describe a pilot study for a two arm, one site randomized controlled trial (RCT) to determine the effect of tele-health monitoring on self-management, quality of life and patient satisfaction. Sixty patients who have been discharged from one acute trust with a primary diagnosis of COPD and who have agreed to receive community clinical support following discharge from acute care will be randomly assigned to one of two groups: (a) Tele-health supported Community COPD Service; or (b) Usual Care. The tele-health supported service involves the patient receiving two home visits with a specialist COPD clinician (nurse or physiotherapist) then participating in daily tele-monitoring over an eight week period. Usual care consists of six home visits to the patient by specialist COPD clinicians again over eight successive weeks. Health status and quality of life data for all participants will be measured at baseline, on discharge from the service and at six months post discharge from the service.

**Discussion:**

The tele-health service under study is a complex service delivered through a collaboration between local authority and health care partners. The implementation of this service demanded significant changes to established working patterns and has been a challenging process requiring considerable planning - a challenge that many providers are likely to face in the future.

**Trial registration:**

Current Controlled Trials ISRCTN68856013

## Background

### COPD

Chronic Obstructive Pulmonary Disease (COPD) is the fifth biggest killer disease in the UK [1]. It is also the second most common cause of emergency admission to hospital and one of the most costly in-patient conditions [2], accounting for £587 m of the £1.08bn spent on hospital admissions for lung disease by the NHS [3]. The current prevalence of COPD in the UK is around 1.5% of the population (approximately 900,000 people), with the condition now being of equivalent incidence in both men and women [4]. The National Institute for Health and Clinical Excellence (NICE) [5] estimates the direct cost of COPD at more than £982 million per year when accounting for indirect costs.

Primarily a smoking related disease, COPD is a long term condition that affects one in six smokers aged over 45 years and is characterized by a chronic, progressive decline in lung function. In the early stages of the disease patients are largely free of symptoms, but as the disease progresses patients may become house-bound, socially isolated and depressed. Patients with COPD thus experience poor quality of life with impaired emotional, social and physical functioning [6]. Exacerbations in symptoms (characterized by increased airway and systemic inflammation and significant deterioration in lung function) occur with increasing frequency and often require hospital management.

### Care delivery model

There is a clear case for the redesign of NHS services for patients with chronic diseases. In 2002, a Cochrane review [5] concluded that a 'hospital at home' approach was a safe and effective option for certain patients with COPD with this recommendation being included within subsequent NICE Guidelines. The NHS Modernisation Agency [7] also proposed that in the future, patients with chronic diseases should no longer occupy acute beds, but rather be treated in a community setting, or supported at home. A key message from the National COPD Patient survey [8] was the need to prioritise personalized COPD planning and self management. Furthermore, in their 2004 annual report [9], the Foundation for Assistive Technology UK (FAST) suggested that chronic disease services needed to be redesigned around patients with a focus on prevention, assessment and self-management.

Tele-health monitoring is defined as the remote exchange of physiological data between a patient at home and medical staff to assist in diagnosis and monitoring. It includes (amongst other things) a home unit to measure and monitor temperature, blood pressure or other vital signs for clinical review at a remote location (for example, a hospital site) using phone lines or wireless technology [10]. The use of community based medical monitoring technology offers real potential for people with long term conditions in that it removes the physical location aspect of health care. Consequently it is believed that effective use of tele-health could lead to significant cost savings [11].

Research has demonstrated that timely and effective treatment and support can shorten COPD exacerbations, leading to a reduction in the need for hospital admission and patient length of stay, resulting in improved outcomes and quality of life for patients [1]. A comprehensive systematic literature review on tele-health for COPD was undertaken by the King's Fund Whole System Demonstrator Action Network [12]. Of the nine COPD studies included in this review, one small randomized controlled trial and three observational studies compared home tele-monitoring and five trials compared telephone support with usual care. Home tele-monitoring and telephone support were found to reduce the rates of re-hospitalization and emergency department visits.

The evidence for community-based tele-health health services is more limited. We are aware of one RCT currently in progress which should be reported upon in 2011 [13]. This is investigating the clinical and cost-effectiveness of using tele-health technology for supported self-monitoring of patients with COPD. This two arm trial is comparing tele-health monitoring with standard care for patients with moderate to severe COPD, recruited from Lothian general practices (GPs) who have been admitted to hospital with an exacerbation of COPD in the previous six months.

No robust studies have been located which examine the use of tele-health monitoring of people in the earlier stages of the condition.

### Local context

The RCT will take place in a region covered by one PCT which has a high incidence of COPD which can be traced to both its coal mining history and the relatively high levels of smoking by the population [14]. It is ranked as one of the most deprived local authorities in the Index of Multiple Deprivation [15]. The high levels of deprivation are associated with poor diet and other adverse lifestyle factors, with smoking being one such factor [16]. The PCT has been recognized as one of the best in the country at detecting COPD by the Eastern Region Public Health Observatory and has been placed in the top 10 for the detection of COPD against all other Primary Care Trusts in England [17].

New service developments have been put in place in the location for this research in order to provide optimal management for people with COPD following hospital discharge. A new Community COPD Service was introduced in May 2009, to manage people with early stage COPD for up to eight weeks following hospital discharge. The remit of the new service is to encourage self-management by patients including education, medication management and lifestyle changes, with the aim of preventing further admissions and improving patient quality of life. In May 2010, the PCT introduced a tele-health supported Community COPD Service. Using the technology, which is personalized to each patient's condition, patients are enabled to monitor their own signs and symptoms, with alerts being provided to community clinicians should any recorded data fall outside expected parameters or if the patient fails to provide data. Thus, community clinicians only need to make additional patient visits to those planned on the care pathway if an alert is raised. Service commissioners and providers identified that this was an opportune time to investigate the impact of tele-health technology compared to traditional methods of delivering post discharge community clinical services. Consequently a pragmatic RCT has been planned with key staff.

## Methods/Design

Given the complexity of the care pathway, the newness of the services and the lack of population-based data on which to base the sample size calculation, in accordance with MRC Guidance, a pilot study will be undertaken. This pilot study will be conducted over a three month period and will operate as a 'mini-trial', testing all elements of the definitive trial. It will use the research methodology developed for the definitive trial in terms of inclusion/exclusion criteria, care pathways, data collection tools and randomisation procedures which are outlined below.

### Design of the Definitive Trial

The definitive study will be a twelve month, single blind, two arm, randomized trial in one Primary Care Trust (PCT) in the United Kingdom. The study population will be patients discharged from acute care diagnosed with COPD who have agreed to use the Community COPD Service as part of their post hospital discharge support.

The study will test the following specific hypotheses:

1. Tele-health monitoring will reduce the proportion of patients with early stage COPD who require further hospital re-admissions to manage their COPD for the duration of, and for six months following discharge from the Tele-health-supported Community COPD Service when compared with those who received the standard Community COPD Service; and

2. Tele-health monitoring will improve the quality of life for patients for the duration of, and for six months following discharge from the Community COPD Service when compared to the standard Community COPD Service.

There are two referral routes into the Community COPD Service:

1. The acute trust employs two COPD nurses who may refer people directly to the Community COPD Service as a supportive early discharge service. Patients not wishing to use the early discharge service will remain under the care of the inpatient COPD nurses until alternative arrangements have been made. Alternatively;

2. Any member of the acute trust nursing staff may use a new telephone referral service for patients with a diagnosis of COPD, who are being discharged from any ward in the hospital. The administration team of the Community COPD Service triage each of these patients to ensure that they are eligible to utilise the service.

### Primary and Secondary Outcome Measures for the Definitive Trial

Two primary outcomes will be measured:

1. The health service primary outcome will be the proportion of patients who are re-admitted to hospital with COPD as the primary or secondary cause of their admission, either during, or six months following their discharge from the Tele-health supported or standard Community COPD Service. This will be assessed through analysis of patient-completed self-reporting diaries, which will be completed both during and for six months following the Tele-health supported or standard Community COPD Service and analysis of hospital admission data.

2. The patient-centred primary outcome will be changes in self-reported health status and quality of life as measured by comparison of the patients' St. Georges Respiratory Questionnaire score upon admission to either the Tele-health supported or standard Community COPD Service, on discharge from the service and six months after discharge from the service.

There are four secondary outcomes:

1. Proportion of patients requiring unscheduled health care support to manage their COPD (including A&E, GP or community nurse visits) either during or six months following the standard Community COPD Service. This will be collected using a paper format self-reporting diary for the standard Community COPD Service and via the monitoring system for the Tele-health supported service; and analysis of hospital, GP and community nursing activity data.

2. Cost effectiveness of intervention. Costs and outcomes will be collected for each individual patient recruited to the pilot trial. The data to be collected for cost estimates will focus on usage of emergency and home-based care by participants. Health related quality of life will be measured using the EuroQol 5 Dimensions questionnaire (EQ-5D), which can be combined with mortality data to produce quality adjusted life years (QALYs). The EQ-5 D is a simple patient completed questionnaire (typically taking less than five minutes to complete), and has been incorporated into the COPD self-completed patient diaries. To ensure monthly data collection it has been embedded into the diaries collected during weeks 1 and 5, whilst the patient is enrolled on the Community COPD Service (both modalities) and subsequent diaries completed each month following discharge from the Community COPD Service.

Data on service usage (for cost analysis) will be accessed where possible from routine data systems of acute and primary care providers and the local authority rather than being reliant upon patient recall. The pilot trial will enable the researchers to examine how feasible it is to both access and analyse this data.

3. Improved self-management of their COPD by the individual patient. This will be assessed through analysis of the results obtained from the St. Georges Respiratory Questionnaire and those from a self-completed patient satisfaction questionnaire devised for this study as described below.

4. Satisfaction with technology as part of their Tele-health supported Community COPD Service. This will be assessed through analysis of a self-completed bespoke tele-health service questionnaire to be completed by patients on the tele-health supported Community COPD Service during their final discharge visit (week 8 after leaving hospital) by Community COPD Service clinical staff.

### Design of the Pilot Study

Given the newness of the Community COPD Service, population-based data upon which to base a sample size calculation for the definitive trial is not yet available. The main purpose of the pilot is therefore to obtain the required data. The PCT Commissioning Directorate for the involved PCT has identified that approximately 1200 patients per year are discharged from the hospital with a primary diagnosis of COPD with the intention being that all of these patients will be offered the standard Community COPD Service provided they meet the eligibility criteria. A preliminary review of patients accepted onto the standard Community COPD Service by the PCT identified that 63% of all patients currently accepted on the programme would be eligible for the trial. If correct, this would suggest that 189 patients would be eligible for randomisation within the pilot timeframe, assuming that consent is forthcoming.

The pilot will afford the opportunity to audit the number of patients referred to, and accepted by, the Community COPD Service, identify the numbers eligible for the trial, and those who consent. For the purposes of this pilot study, a conservative view of the PCT estimate has been taken, resulting in a recruitment target of 60 patients over a three month period.

This pilot will be used to:

1) Obtain data to calculate the sample size for the definitive trial, including:

• recruitment

• retention and

• adherence rates;

2) Confirm the integrity of the study protocol, including:

• Inclusion/exclusion criteria;

• Fidelity of the care pathways;

• Installation, recovery, cleaning and re-stocking of tele-health equipment; and

• Staff training.

3) Confirm the utility of data collection forms and questionnaires;

4) Confirm the randomization procedure;

5) Confirm recruitment rates and consent procedures;

6) Confirm the acceptability of the intervention to the patients; and

7) Confirm the data collected will allow for reporting upon the identified outcome measures.

Assuming that no issues are identified during the internal pilot phase and that approval has been received from the South Yorkshire Research Ethics Committee, the definitive trial will commence immediately following from the pilot.

### Ethics

A favourable ethical opinion for this research proposal has been received from the South Yorkshire Research Ethics Committee (reference: 10/H130/48).

Participants will only be recruited to the trial if they can provide informed consent. The Department of Health [18] state that "for consent to be valid, it must be given voluntarily by an appropriately informed person who has the capacity to consent to the intervention in question". To ensure that this is the case in this study, the following measures have been implemented.

#### Mental capacity

The Mental Capacity Act 2005 [19] defines a person who lacks capacity as "a person who is unable to make a decision for themselves because of an impairment or disturbance in the functioning of their mind or brain". It goes on to state that "a person must be assumed to have capacity unless it is established that they lack capacity. If there is any doubt, then the healthcare professional should assess the capacity of the patient to take the decision in question". For this study, all patients will be initially assessed by health care professionals who will then provide details of potential participants to the researchers.

#### Voluntary consent

Department of Health guidance [18] states that for consent to be valid "it must be given voluntarily and freely, without pressure or undue influence being exerted upon them". Every care will be taken to ensure that the patient is able to give voluntary consent and they will have the option to deny consent or withdraw from the research at any time and without providing any reason or explanation.

#### Sufficient information

Department of Health guidance [18] states that "to give valid consent, the person needs to understand the nature and purpose of the procedure. Any misrepresentation of these elements will invalidate consent". When considering what information to provide, the Act states that the researcher should "try to ensure that the person is able to make an informed judgement on whether to give or withhold consent". To this end, a detailed information sheet has been developed for the potential research participants who will be asked to participate in the trial. The information sheet provides contact details for obtaining further information, details on the planned research, rights to non−participation, how their details were selected and how data will be stored according to the principles of the Data Protection Act [20].

#### Timing of consent

Department of Health guidance [18] states that "it is good practice where possible to seek the person's consent to the proposed procedure well in advance, when there is time to respond to the person's questions and provide adequate information". Consequently hospital nursing staff will provide the patient with the information sheets prior to discharge which they can review at their leisure over a period of no less than 24 hours before a specialist COPD clinician visits them to undertake their first clinical assessment, at which time the trial will be explained and their consent to participate in the trial will be sought.

#### Form of consent

Although completion of a consent form is in most cases not a legal requirement it is recognized as good practice, so written consent will always be obtained. Copies of the consent forms to be used are included in the appendices.

### Patient Population and Recruitment

#### Inclusion criteria

• Open to male and female adult participants (> age 16);

• Being discharged from the acute care Trust and diagnosed with COPD;

• Between 1 and 3 previous admissions (including the current admission) in the previous 12 months according to the hospital discharge abstract *from the current date of discharge *where COPD is the primary or secondary documented reason for hospitalisation

• Patient has consented to be included in the caseload of the Community COPD Service;

• Willing to use Tele-health technology as part of their discharge plan;

• Able to communicate in English and read English (a requirement of the technology); and

• Have a telephone landline and a viable telecommunications network with no more than three internal telephone extensions.

#### Exclusion criteria

• Prior/current involvement in another tele-health initiative;

• Cognitive impairment to the extent that it impedes ability to participate;

• Other significant impairments which will restrict ability to participate;

• No telephone landline;

• Unwilling to use Tele-health technology;

• Existence of co-morbidities which require on-going intervention from other community nursing services;

• More than three hospital admissions within twelve months of the date of discharge for which COPD is the primary diagnosis; and

• Patient unable or unwilling to provide written or oral informed consent.

**Figure 1:**
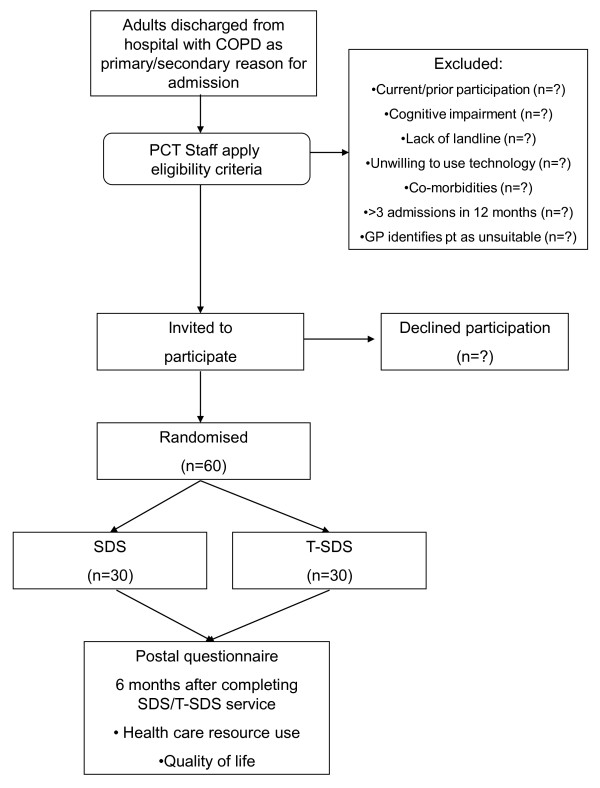
**Graphical depiction of the interventions**.

Basic details (age, gender, prior hospitalisations for COPD) will be collected for all eligible patients to allow completion of a CONSORT [21] flow chart as depicted in Figure 1.

Whilst still in hospital, either the COPD or ward staff (depending upon the referral route) will provide the patient with a leaflet about the Community COPD Service, a patient information sheet and a copy of the research consent form prior to their discharge. The patient will be made aware that the clinicians from the Community COPD Service will provide them with more information about the research on their first visit (day after patient's discharge from the hospital). On this first home visit, a clinician from the Community COPD Service, who has undertaken Good Clinical Practice training and is therefore qualified to seek patient consent to participate in the trial, will confirm that patients meet all the inclusion criteria to participate in the trial; will explain the study if any additional information is requested; and seek consent to participate in the trial. They will then aim to recruit patients initially to the service and, secondly, to the trial. Should a patient not wish to participate in the research they can still receive the standard Community COPD Service but will not be included in the study. As identified in the exclusion criteria, any patient unwilling to consider the technology as part of their care plan will be excluded from the trial although they can receive the standard Community COPD Service.

### Randomisation

Consented patients will be randomized using a web-based system developed by the University of Sheffield Clinical Trials Research Unit. A simple randomisation sequence will be used to allocate patients to either the standard Community COPD Service or the Tele-health supported Community COPD Service. It is impossible to blind patients or clinical staff to the trial arm, but research staff will be blind to the allocation. Administration staff at the Community COPD Service will use the web-system to identify the allocation for the next patient who consents to participate in the trial, but at this stage the clinician seeking consent from the patients will be blinded to the allocation to prevent any bias in recruitment. Once consent has been received, the clinician will contact the administration centre to obtain the allocation. At this point they will inform the patient of the allocation and tell them whether they will receive the tele-health supported or standard community COPD service.

### Intervention

**Figure 2:**
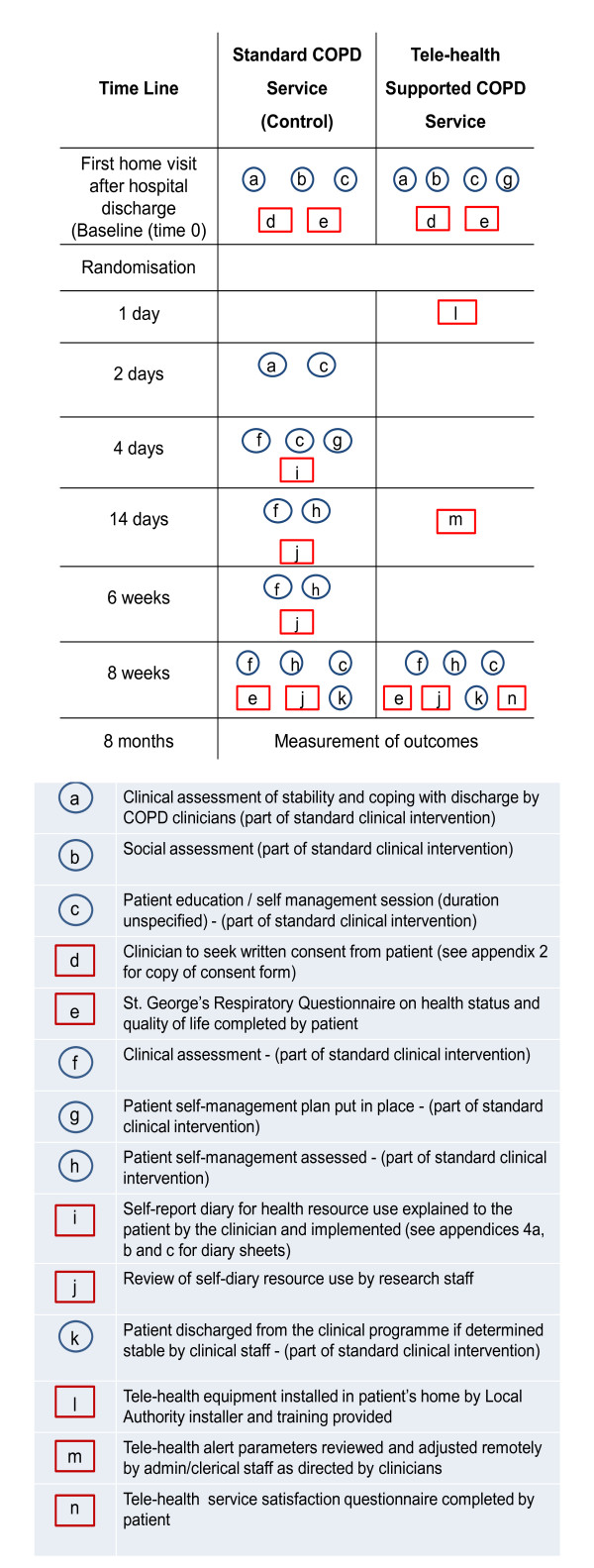
**CONSORT Flow Chart**.

The two care pathways and associated research interventions are depicted graphically in Figure 2. All visits and telephone calls described below will be undertaken by clinical staff of the Community COPD Service team.

#### Tele-health Intervention

Patients recruited to the Tele-health supported Community COPD Service will be taught how to use the technology to aid monitoring of their condition. The patient will be required to enter information into the tele-health equipment on a daily basis to monitor their condition. Data provided to the system will be recorded on the web server so patient compliance with using the equipment can be assessed. If the patient fails to enter information into the equipment for three continuous days the system generates an alert on the web server which is acted upon by the COPD team.

#### Usual Care Group

Patients recruited to the standard Community COPD Service will not receive the technology but will have six visits with specialist COPD clinicians from the Community COPD Service as described in Figure 2.

### Data collection tools

#### St. Georges Respiratory Questionnaire

Patients will be asked to complete the St. Georges Respiratory Questionnaire score upon admission to either the Tele-health supported or standard Community COPD Service, on discharge from the service and six months after discharge from the service. This questionnaire is a validated and widely-used instrument which measures health impairment in patients with COPD on a scale of 0 to 100 (greatest impairment). This scale is responsive to change with a minimum important difference (MID) of 4.

#### Diaries

Patients recruited to the Usual Care Group will track their use of health services to assist in the management of their COPD by means of a self-reported questionnaire whilst on the eight week programme and for the subsequent six months. Patients will record whether they have contacted a health service provider because they had concerns about their COPD, who they contacted, why they contacted them and what happened as a result (telephone consultation, visit). The diaries will also be used to capture EQ5 D information on problems with mobility, self-care, usual activities, pain/discomfort and anxiety/depression.

Patients on the Tele-health supported Community COPD Service will be asked to answer the same questions as part of their routine use of the technology.

#### Tele-health service questionnaire

Patients on the Tele-health supported Community COPD Service will be asked to complete a questionnaire on discharge from the service. The questionnaire will ascertain their opinions about the technology, the installation process, training, use of the equipment, availability of support and removal of the equipment from their home.

### Research Governance

The trial will be conducted in accordance with MRC Guidelines [22,23]. The acute trust will act as the sponsor of this project.

A Trial Management Committee will be established and will consist of the Principal Investigator, Research Project Manager, research staff, PCT Operations Manager, Operational Project Manager and the clinicians. Meetings of this team will be held monthly during the three month pilot.

A Data Monitoring Committee will meet towards the end of the pilot to review adherence to enrolment, randomisation strategy and examine the trial data. This committee will consist of an independent statistician, an independent trialist, the Principal Investigator and Research Project Manager.

### Patient and Public Involvement (PPI)

In the acute trust, a local group founded on the principles of INVOLVE [24], known as the Consumers Research Advisory Group (CRAG), meet three to four times per year, and their key role is undertaking reviews of proposed projects. This generally involves reading research proposals, and other project literature, and providing feedback on any areas which the researcher has requested, or those the group feel are in need of revision or clarification. A copy of the protocol for this pilot has been shared with CRAG and will be discussed at their next meeting. Feedback from the group will be incorporated into the design and conduct of the subsequent research phases. The protocol for the definitive trial will again be shared with this group as will the findings of the research.

## Discussion

The high local prevalence of COPD contributed to the £2.2 million cost of related admissions billed to the local health service between April 2006 and March 2007 [16]. Consequently, the acute trust, the Commissioners and PCT have all recognized the urgent need to support patients and minimize unnecessary hospital admissions and emergency room visits. This initiative is one of the approaches being considered to address this goal.

Whilst the need to improve care delivery modalities and reduce reliance on the acute sector is recognized, in reality, the shift to the primary sector is proving more difficult to enact than initially anticipated. This project is posing a number of challenges for the PCT undertaking the implementation of the tele-health supported Community COPD Service.

In developing their project plan for implementation of the tele-health supported service, the PCT realized that they did not have the resources and expertise to undertake the installation of equipment into patient's homes. Within the city, the Local Authority had established a service providing a 24-hour, 365 day a year emergency response service for approximately 7,000 residents of the Borough, with approximately 4,700 service users linked to the service using Individual Alarm Units and the remainder in Local Authority and Housing Association accommodation linked by hard-wired intercom systems. Clearly this centre had experience in installing comparable technology into people's homes, so the PCT created a partnership with the Local Authority, tasking them with the installation of the tele-health equipment within 72 hours of hospital discharge.

This horizontal integration is an innovative model which has needed detailed planning and a great deal of collaboration. Both partners have provided extensive support to the project, developing detailed flowcharts to identify key activities, responsibilities and timings to ensure all elements of the project remain on track; developing training materials for staff and patients; shared forms to capture data required by all partners and shared spreadsheets to track patient-specific data to ensure there are no gaps in service delivery and technology-related data to ensure that its location and condition (installed and in use/in stock/unavailable - awaiting cleaning) are always known. Methods to address requirements for routine portable appliance (PAT) testing to meet the Electricity at Work Regulations [25], infection control, confidentiality of patient records and the safe installation of the technology have all needed to be addressed.

Tele-health may offer a way to provide care and encourage self-management of the condition for early stage COPD patients within the primary care sector, and this will be evaluated in the planned RCT. However, preparation for this RCT has provided evidence of specific challenges in implementing tele-health supported services which may require quite innovative solutions.

## Competing interests

The authors declare that they have no competing interests.

## Authors' contributions

DAF participated in the design of the study, developed the protocol, obtained approvals from the South Yorkshire NHS Research Ethics Committee and the local Research Governance Committee and drafted the manuscript. JK undertook patient interviews to test the data collection tools used in the study. MH participated in the design of the study. GAM participated in the design of the study and participated in the development of the protocol. All authors read and approved the final manuscript.
